# Electrochemical Determination of Chlorogenic Acid in Nutraceuticals Using Voltammetric Sensors Based on Screen-Printed Carbon Electrode Modified with Graphene and Gold Nanoparticles

**DOI:** 10.3390/ijms22168897

**Published:** 2021-08-18

**Authors:** Irina-Georgiana Munteanu, Constantin Apetrei

**Affiliations:** Department of Chemistry, Physics and Environment, Faculty of Sciences and Environment, “Dunărea de Jos” University of Galaţi, 800008 Galaţi, Romania; georgiana.munteanu@ugal.ro

**Keywords:** chlorogenic acid, cyclic voltammetry, graphene, gold nanoparticles, sensor

## Abstract

The present study describes the electrochemical properties of three screen-printed electrodes (SPEs), the first electrode being carbon-based (C), the second graphene-based (GPH), and the third based on GPH modified with gold nanoparticles (GNP). These electrodes were used for the study of the electrochemical behavior of chlorogenic acid in different aqueous solutions, at pH = 7. In chlorogenic acid solution, a redox process was noticed in the case of all three electrodes; GPH and GNP significantly improved the sensor response regarding sensitivity and reversibility, a fact demonstrated by characterizing the sensor by cyclic voltammetry in potassium ferrocyanide, which corresponds to the exchange of two electrons and two protons. Moreover, the calibration curves for each sensor were developed, subsequently calculating the detection limits (LOD) and the quantification limits (LOQ). Low LOD and LOQ were obtained, the best—of the order of 10^−7^ M (LOD = 0.62 × 10^−7^ M; LOQ = 1.97 × 10^−7^ M)—being obtained in the case of GPH-GNP-SPE, which demonstrates that the method may be applied for determining chlorogenic acid in real samples. Thus, the sensors were successfully used for the quantitative determination of chlorogenic acid in three nutraceutical products. The validation of the results was done using the FTIR method. The results obtained by cyclic voltammetry were in accordance with those obtained by the spectrometric method, without significant differences from a statistical point of view.

## 1. Introduction

Phenolic compounds represent an important class of compounds because of their antioxidation activity, having anti-inflammatory, anti-carcinogenic, and anti-microbial properties [1]. They are classified in two main categories: benzoic acids and cinnamic acids. These compounds exist, mainly, under the form of hydroxybenzoic or hydroxycinnamic acids and may be present in free form as well as in conjugated form.

Hydroxycinnamic acids—such as chlorogenic acid, p-coumaric acid, caffeic acid, ferulic acid, and synaptic acid—act as antioxidant compounds that also have various roles in biological systems. The most relevant mechanism for antioxidant action is presupposed to be linked to their capacity to neutralize free radicals, depending on the ability to donate hydrogen or release electrons, and on the stability of the resulting phenoxyl radicals [2]. In addition to these, other action mechanisms were described, from among those worth mentioning are chelating transitional materials—for example, iron and copper (well-known catalysts of oxidative stress), inhibiting the enzymes that generate ROS/RNS (reactive oxygen species/reactive nitrogen species) and modulating gene expression [3].

**Figure 1:**
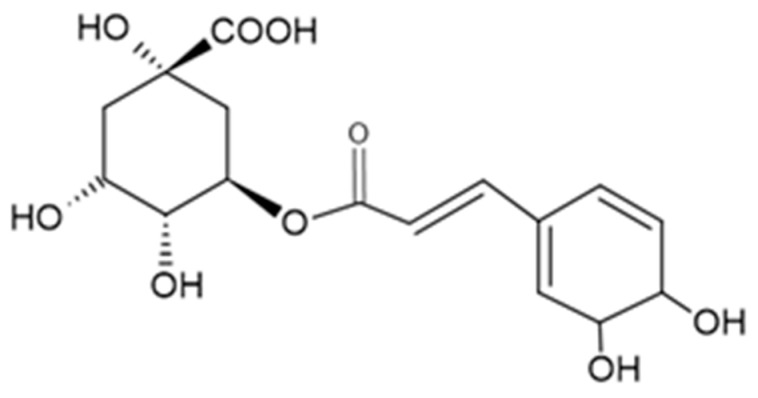
Chemical structure of CGA.

Chlorogenic acid (CGA), (1S, 3R, 4R, 5R)-3-[(E)-3-(3,4-dihydroxyphenyl) prop-2-enoil] oxi-1,4,5-trihydroxycyclohexane-1-carboxylic acid, is the ester of caffeic acid and of quinic acid, a phenolic natural compound whose structure is presented in Figure 1. Being a bioactive compound with many therapeutic effects (antihypertension, anti-inflammatory, adjuvant in preventing obesity, antitumoral, adjuvant in preventing neurodegenerative diseases) [4], CGA may be found in more than 170 types of Chinese pharmaceutical products in the form of capsules, tablets, or injectable products based on plants. Thus, a precise and rapid determination of CGA in pharmaceutical products is of great importance [5].

Therefore, many methods for determining CGA, as well as for its derivatives out of coffee beans and other plants, were elaborated. The most frequently used methods are spectroscopy in infrared [6], high performance liquid chromatography (HPLC) [7], capillary electrophoresis [7,8,9], liquid chromatography–mass spectrometry with electrospray ionization [10], chemiluminescence [11], and micellar electrokinetic chromatography [12]. Although these methods proved to be efficient for quantifying CGA and its derived products, a number of deficiencies were identified: they are time consuming, laborious, and require expensive instruments [13].

In recent years, electrochemical methods for determining phenolic compounds were developed and investigated on a large scale, having a series of advantages, such as simplicity, high sensitivity, reproductivity, rapid response, and reduced costs [13,14]. In addition to their application in fundamental studies of oxidation and reduction processes for discovering reaction mechanisms, these techniques are also used in studying kinetic and thermodynamic processes of transferring ions and electrons [15]. One of these methods, cyclic voltammetry (CV), is used on a large scale for the study of redox processes [16], particularly for obtaining information regarding the nature of intermediary products and the stability of reaction products [17].

Until now, for the electrochemical determination of CGA, electrodes modified with modifiers—such as ionic liquid that contains iridium nanoparticles and polyphenol oxidase [18], horseradish peroxidase [19], bean sprout homogenate and chitosan microsphere composite [20], tetranuclear copper complex (II) [Cu_2_(µ-OH)(bpbpmp-NO_2_)]_2_[ClO_4_]_2_ [21], molecularly printed siloxane [22], and a DNA compound/silicon-titanium [19]—were reported.

Developing and characterizing new methods of electroanalysis may be improved by using SPEs [23]. Hart, Banks, and Wang have studied the use of SPEs for large-scale production and have reached the conclusion that these devices have a series of advantages, such as ease-of-use [24], portability, and reduced costs for analysis [25].

The SPEs can be selected according to the material from which the working electrode is made, for example carbon, gold, platinum, or other metals, depending on the ongoing study. Due to the high reproducibility between electrodes, these sensors are suitable for a wide range of applications: clinical sensing platforms development and environmental and food industry analysis. Moreover, sensor arrays allow the determination of multiple substances in parallel [26].

Compared with the traditional methods used to determine certain compounds, the great versatility of SPEs is due to the wide range of strategies in which the working electrode surface can be modified [27]. In addition to the possibility of using a wide variety of compounds to change the composition in order to control the electrochemical behavior, different strategies for modifying the surface of SPEs have allowed the employment of these electrodes in a variety of sensors and biosensors [28]. Another key factor of SPE modification was the appearance of a great variety of nanomaterials. Due to the many advantages related to their physical and chemical properties, these nanomaterials have demonstrated new ways of interacting with biological elements and have been widely used in the development of biosensors [29].

In addition, using nanomaterials as a sensitive elements and especially carbon-based nanomaterials [30] and metallic nanoparticles may improve the performance characteristics of SPEs [31].

In the past decades, nanostructured materials have aroused dramatic interests and have become an intensive research area due to their high specific surface area, finite small size, high porosity, and unique physical and chemical properties [32].

GPH was studied on a large scale because of its properties and its advantages, such as a large active surface that improves the absorption capacity of the organic molecules and very good electric conductivity [33].

Nanoparticle-based technologies have contributed to the development of a new generation of detection tools. Taking into account their distinct optical, chemical, electrical, and catalytic properties, GNPs have been extensively studied for biological and chemical detection and also for analytical applications [34].

GNPs allow the direct transfer of electrons between the redox compounds and the materials of the electrode, detecting the signal being done without the need of transferring mediating electrodes [35]. Furthermore, this direct transfer is facilitated by characteristics of GNPs, such as the high ratio between surface and volume, energy increased by the surface, and the capacity to function as electro-conductive paths between protein groups and the surface of the electrode [36].

Due to the ease of functionalization of the GNPs surface, researchers have been able to create various functionalities for a range of applications [37]. Gold nanoparticles are of major importance in the biomedical field, applicable to the development of vaccines, the administration of medicines, and the development of immunoassays [38].

Developing new sensors based on GPH and GNP is a promising way to achieve the simple and rapid detection of various analysts, including CGA. The developed sensors remarkably enhanced the electrocatalytic response towards redox behavior of CGA [39]. On comparing the sensitivity of modified sensors with bare C-SPE, it was observed that a film of nanogold exhibited increased electrocatalytic response and quantification of CGA [40]. Consequently, the purpose of this study was to develop, optimize, and validate the electroanalytical method based on cyclic voltammetry for evaluating and quantifying CGA in various nutraceutical products using voltammetric GPH- and GNP-based sensors [41].

## 2. Results and Discussions

### 2.1. Preliminary Studies

The behavior of the three electrodes—C-SPE, GPH-SPE, and GPH-GNP-SPE—in a 10^−1^ M PBS solution, at pH = 7, optimum potential range between −0.4 V and +1.3 V, was studied during the first stage. Following the measurements, no peaks were registered in the potential field analyzed, which demonstrates the high purity of the materials used for the electrodes and the lack of active surface contamination. It was noticed that, in the case of the GPH-GNP-SPE electrode, the base currents were reduced, which represents an advantage of modifying the sensor surface with GPH and GNPs.

### 2.2. Electrochemical Properties of the Electrodes in Electroactive Solution

During the next stage, the electrochemical behavior of SPEs in a solution containing 10^−3^ M K_4_[Fe(CN)_6_] dissolved in 10^−1^ M PBS, pH = 7, was analyzed using cyclic voltammetry as the detection method [42]. The potential field in which the signal proved to be stable was between –0.4 and +0.7 V. In view of stabilizing the sensors, 5 cycles were registered in the solution to be analyzed.

**Figure 2:**
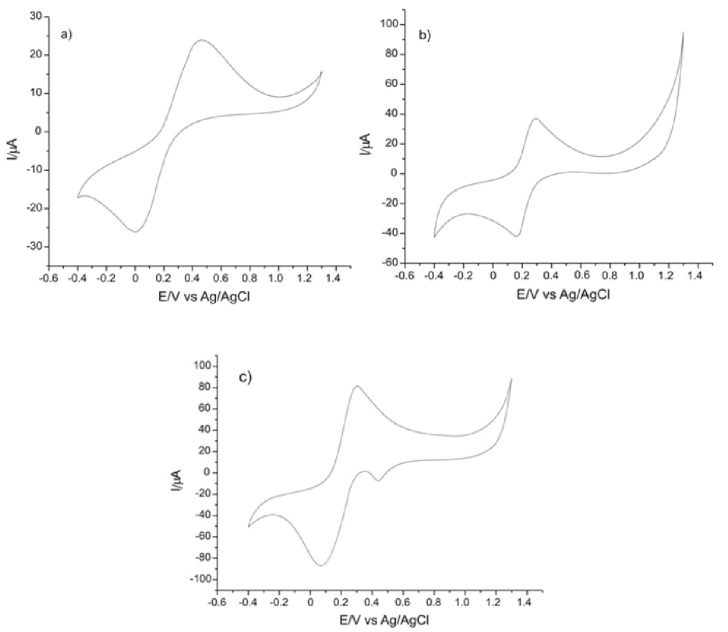
The cyclic voltammograms (CVs) of (**a**) C-SPEs, (**b**) GPH-SPE, and (**c**) GPH-GNP-SPE immersed in 10^−3^ M K_4_[Fe(CN)_6_]—PBS solution. Scan rate 0.1 V·s^−1^.

Figure 2 shows a pair of well-defined peaks, an anodic and a cathodic one, for C-SPE and GPH-SPE. Additionally, in the case of GPH-GNP-SPE, two peaks of low intensity are noticed (an anodic and a cathodic one). The first pair of peaks is due to the reversible oxidation of the ferrocyanide ion to ferricyanide. For the sensor modified with gold nanoparticles, the occurrence of the second pair of peaks may be explained by the presence of the modifying nanomaterial on the sensor surface. All three electrodes registered well-defined and reproducible peaks, allowing use in further analyses.

The main parameters obtained from CVs are shown in Table 1.

It was noticed that for C-SPE and for GPH-SPE, E½ had approximately the same value. This was close to the E½ value obtained for GPH-GNP-SPE. The ratio Ipc/Ipa was close to the ideal value of 1 in all three cases. The GPH-GNP-SPE electrode showed the highest rate of reversibility, the separation between the anodic and cathodic peaks being smaller than in the case of the other electrodes, and the Ipc/Ipa ratio was >1 (1.07). This demonstrates that the reaction process in the case of the electrode modified with gold nanoparticles was quasi-reversible. All three electrodes showed a similar electrochemical behavior, according to the parameters obtained, allowing successful use in further determinations.

The highest current values were obtained for GPH-GNP-SPE, with values close to those obtained for GPH-SPE and somehow different from those obtained for C-SPE. Taking these results into account, the greatest sensitivity in detecting the ferrocyanide ion was obtained in the case of GPH-GNP-SPE (where the values for Ipa and Ipc are the highest).

**Figure 3:**
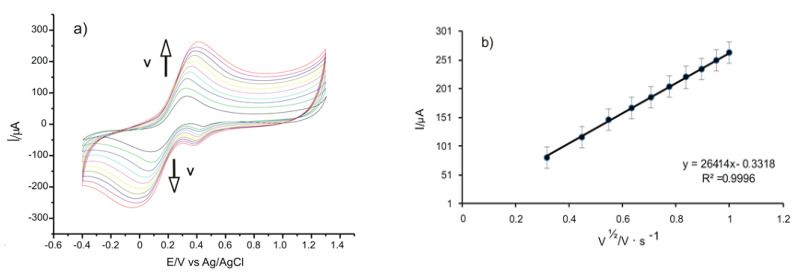
(**a**) CVs of GPH-GNP-SPE immersed in 10^−3^ M K_4_[Fe(CN)_6_]–10^−1^ M PBS solution registered with scan rates in the range of 0.1–1.0 V·s^−1^. (**b**) Plot of the linear dependence between the anodic peak current and the scan rates.

To study the kinetics of the processes, the CVs of the sensors at various scan rates, between 0.1 and 1.0 V·s^−1^, were recorded, this being useful in estimating the active surface of SPEs. For determination, a double 10^−3^ M potassium ferrocyanide solution—10^−1^ M PBS solution of pH = 7—was used. The results obtained in the case of GPH-GNP-SPE are presented in Figure 3.

Figure 3a shows that the increase of the peak currents is directly proportional with the increase of the scanning rate, with a slight shifting of the anodic peak potential to the right and to the left of the cathodic peak potential.

To check the determining factor of the oxidation-reduction process, models of linear regression were developed, correlating Ipa with the scanning rate or with the square root of the scanning rate. In the present case, as shown in Figure 3b, a linear dependency between Ipa and the square root of the scanning rate, I(A) = 0.0001 × v^1/2^ (V·s^−1^)^1/2^ + 1 × 10^−5^ (R^2^ = 0.9996), is noticed. This linear dependency suggests that the oxidation-reduction of the potassium ferrocyanide is controlled by the diffusion of the electroactive species, this being the determining stage in the electrochemical process.

Table 2 presents the values obtained for the linear regression equation, the determination coefficient (R^2^), active surface areas, geometrical areas, and the roughness factor in the case of all SPEs studied.

The Randles–Sevcik equation [43] was used to calculate the active areas of the electrodes, employing the linear dependency between Ipa and v^1/2^.

Ipa = 268,600 × n^3/2^ × A × D^1/2^ × C × v^1/2^ (1)

where Ipa = the anodic peak current measured in amperes, n = no. of electrons transferred following the redox process, A = electrode area, in cm^2^, D = diffusion coefficient, in cm^2^/s, C = concentration, in mol/cm^3^, and v = scanning rate, in V/s. For the potassium ferrocyanide, the diffusion coefficient was D = 7.26 × 10^−6^ cm^2^·s^−1^ [43].

The active area of the electrodes can be calculated from the slope of the Ipa equation, depending on v^1/2^. The area of the geometric surface of the electrodes was 0.1256 cm^2^.

The results obtained for the values of the studied electrode active surface areas are also given in Table 2. It can be noticed that the highest value was obtained for GPH-GNP-SPE, followed by GPH-SPE, while the lowest value was obtained for the C-SPE area. These results are in keeping with the intensities of the peaks. The ratio between the area electrochemically determined (active area) and the geometric area represents the roughness factor. Table 2 indicates that the highest value of the rugosity factor was obtained in the case of GPH-GNP-SPE, due to GPH and the gold nanoparticles, which increase the active surface of the sensor, as well as due to their synergic effects [22].

According to the results obtained, it may be concluded that GPH-GNP-SPE was the most sensitive, followed by GPH-SPE and C-SPE. Nevertheless, all three electrodes may be used successfully to determine phenolic compounds such as CGA.

### 2.3. Electrochemical Responses of Sensors in CGA Solution

The electrochemical behavior of sensors in CGA solution was studied by means of cyclic voltammetry.

The CVs of the 10^−3^ M CGA solution were recorded. The 10^−1^ M (pH = 7) PBS solution was used as supporting electrolyte. The value of the solution pH has considerable influence in the electrochemical study of phenolic compounds [44]. In keeping with the literature, a value 7 of the pH is optimum in the case of phenolic compounds [22]. Consequently, this pH value was used in further determinations.

**Figure 4:**
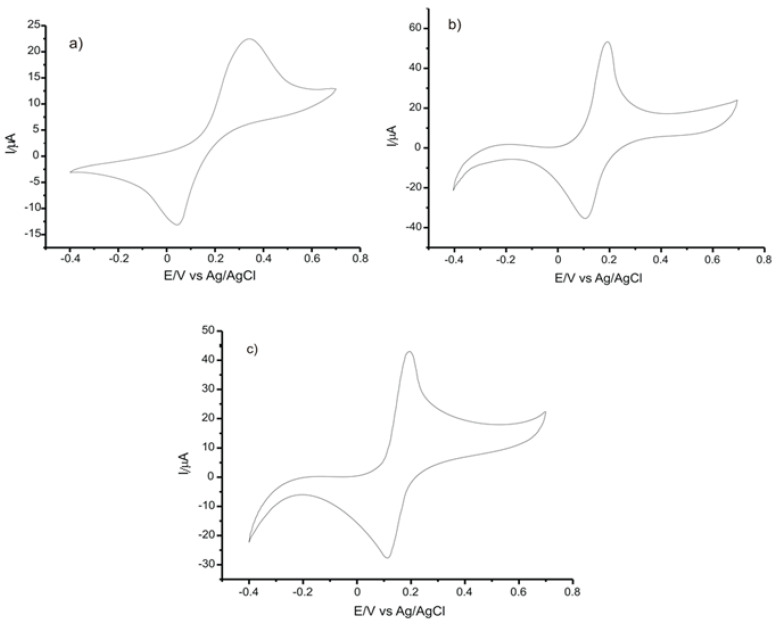
CVs of of (**a**) C-SPEs, (**b**) GPH-SPE, and (**c**) GPH-GNP-SPE immersed in 10^−3^ M CGA solution (support electrolyte 10^−1^ M PBS solution). Scan rate 0.1 V·s^−1^.

To obtain a stable answer from the sensor, five cycles were necessary in the optimized potential field (from −0.4 V to +0.7 V). The CVs presented in Figure 4 were obtained after signal stabilization.

What may be noticed in all three CVs is the occurrence of an anodic peak corresponding to the oxidation process and of a cathodic peak corresponding to the reduction process. Since the CGA contains two OH groups in orto position, by oxidating this compound, the respective quinone is formed, releasing two electrons and two protons [22].

**Figure 5:**
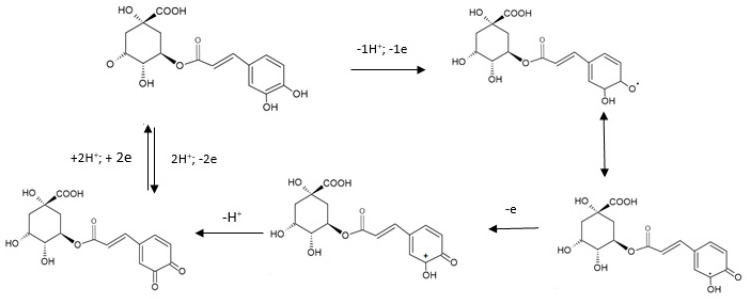
The oxidation mechanism of CGA.

The electrochemical electrooxidation of the CGA presupposes, initially, the pre-dissociation of a proton, with the formation of a monoanionic species. The latter is in turn oxidated to generate a radical. This radical formed is subjected to a new rapid electronic transfer, with the formation of a carbocation which, through dehydration, forms the respective quinone. During the reversed scanning, the quinone formed is reduced to CGA. The oxidation mechanism is presented in Figure 5.

In the case of the sensors studied, a single redox process was noticed; it presupposes the exchange of two electrons and two protons in one stage, with the formation of the quinone derivative.

Table 3 illustrates the most important parameters obtained from the CVs or calculated depending on the experimental parameters.

All three electrodes studied showed two clear, well-defined peaks and a quasi-reversible behavior, as indicated by the ΔE (V) value and the Ipc/Ipa ratio. The GPH-SPE electrode had the most intense anodic and cathodic peaks, highlighting the best sensitivity in detecting CGA. The peaks noticed in the case of GPH-GNP-SPE are more intense by comparison with those recorded for C-SPE, which demonstrates that the electrochemical processes are facilitated by modifying sensors with nanomaterials.

**Figure 6:**
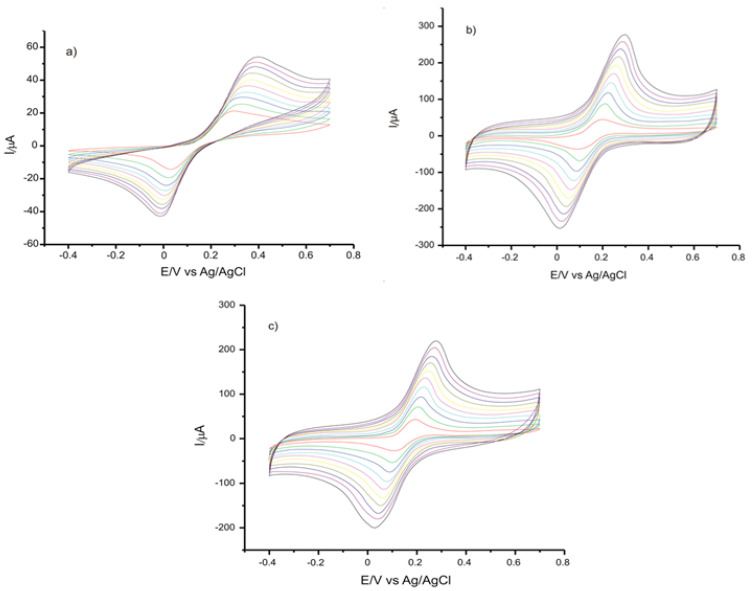
CVs of (**a**) C-SPE, (**b**) GPH-SPE, (**c**) GPH-GNP-SPE immersed in 10^−3^ M CGA solution, recorded at scan rates between 0.1 and 1.0 V·s^−1^.

Figure 6 presents the CVs of the three sensors studied, immersed in CGA 10^−3^ M solution, at scan rates ranging from 0.1 to 1.0 V·s^−1^.

To confirm that the oxidation of CGA is a process controlled by diffusion (according to the Randles–Sevcik equation), the kinetics of the peaks, depending on the square root of the scanning rate, was graphically represented. As may be noticed, there is a linear dependency with v^1/2^, which demonstrates that the process is controlled by diffusion in the case of each sensor used.

The Randles–Sevcik equation allows the calculation of the diffusion coefficient of the CGA for each screen-printed electrode used. The values obtained are presented in Table 4.

The D values obtained for the CGA with SPEs are comparable with those obtained with other carbon-based sensors reported in the literature [32,33]. Metallic nanomaterials, particularly gold nanoparticles, have the capacity to facilitate the transfer of electrons due to their excellent conductivity [34,36], having a large surface-to-volume ratio and a very good biological compatibility [36].

### 2.4. Calibration Curve and Determination of Detection Limit

The CVs in CGA solutions of various concentrations were recorded using C-SPE, GPH-SPE, and GPH-GNP-SPE. The calibration curves were achieved in the concentration field of 0.1–1.20 µM. The 10^−1^ M PBS (pH = 7) solution was used as supporting electrolyte, and the CGA stock solution had a concentration of 10^−3^ M. The scanning rate was 0.1 V·s^−1^ in each case, and the potential field was situated between −0.4 V and +0.7 V.

**Figure 7:**
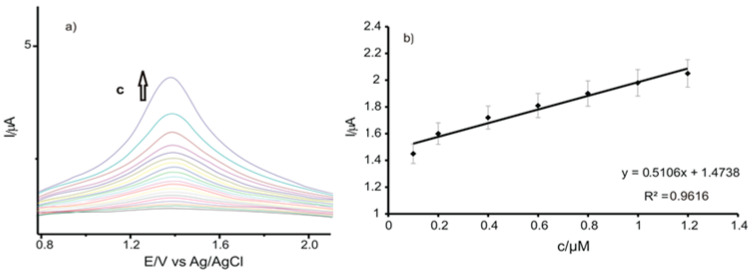
(**a**) Zoomed-in view of the anodic peak zone of the CVs registered with GPH-GNP-SPE immersed in CGA solutions with the concentrations in the range 0.1–1.20 μM; (**b**) linear dependence between the anodic peak current and the concentration of the CGA solution.

The variation of the sensor response depending on the concentration of the CGA solution is presented in Figure 7a. As may be seen, the intensity of the anodic and cathodic peaks increases with the increasing concentration.

Figure 7b illustrates the linear dependency between the anodic peak current and the CGA concentration in the field of 0.1–1.20 µM when using GPH-GNP-SPE. For higher concentrations, the electrochemical signal increases more slowly due to the saturation of the active centers on the surface of the working electrode.

The limits of detection (LOD) and the limits of quantification (LOQ) were calculated using the following equations [45]:

LOD = 3σ/m; LOQ = 10σ/m(2)

where σ is the standard deviation (SD) of the electrochemical signal for the blank sample for the potential corresponding to CGA, and m is the slope of the calibration curve.

Table 5 shows the results obtained for LOD and LOQ, calculated for the three sensors used in this study.

The low values of the detection limit and of the quantification limit are due to the increased sensitivity of the sensors used in this study, which demonstrates the feasibility of the voltammetric method for the CGA analysis with real samples, such as various pharmaceutical products.

The lowest detection limit was obtained for GPH-GNP-SPE. Consequently, the sensitivity of the sensors decreased in the following order: GPH-GNP-SPE > GPH-SPE > C-SPE.

The values obtained for LOD were compared with those reported in literature, as shown in Table 6.

The GPH-GNP-SPE sensor generally had a lower LOD than the majority of sensors reported in the literature. The LOD and LOQ values of the GPH-GNP-SPE sensor were acceptable for detecting CGA in real samples. Therefore, this sensor was used to determine the CGA in nutraceutical products.

It is worth mentioning that graphene dispersed multi-walled carbon nanotubes composite modifiers for glassy carbon electrodes, molecularly imprinted siloxanes on gold electrodes or imidazolium derivatives modified screen-printed electrodes are more or less similar with the metallic nanoparticles or graphene-based modifiers for screen-printed carbon electrodes from analytical point of view. Advantages related to the GPH-GNP-SPE are also attributed to its reproducible synthesis, on large areas, characteristic conversely unattained when an electrode is modified with polymers, dispersions of nanoparticles, etc., through drop casting, adsorption, and under different working conditions (room temperature, humidity, etc.) [50].

### 2.5. Quantitative Determination of CGA in Nutraceutical Products

The following analysis consisted in determining CGA from real samples, with three commercial products being selected—namely, Green Coffee Extract (Rotta Natura), Green Coffee (Pro Natura), and Green Coffee Fit (Pro Natura). These products were analyzed using two methods: CV (the method described in this study) and FTIR analysis (standard method) [51]. Therefore, the goal of this analysis was to compare the results obtained through the methods mentioned and to validate the electroanalytical method.

**Figure 8:**
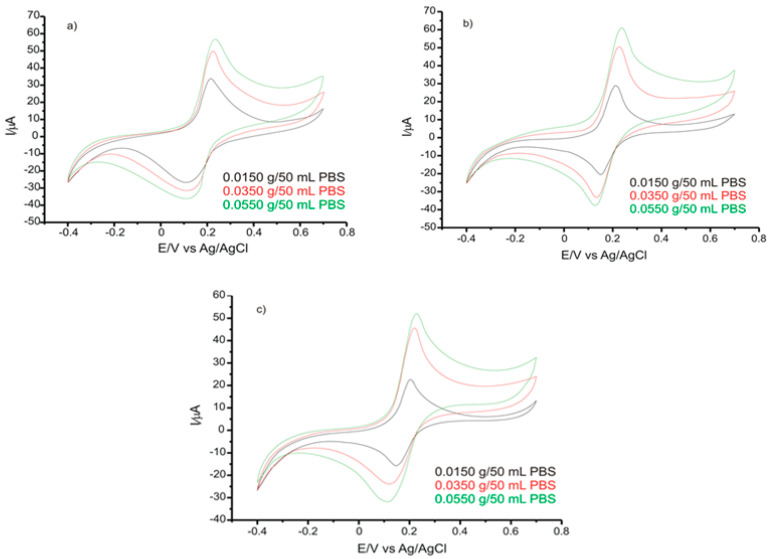
CVs of GPH-GNP-SPE immersed in solution with different concentrations prepared from (**a**) Green Coffee Extract (Rotta Natura); (**b**) Green Coffee (Pro Natura); (**c**) Green Coffee Fit (Pro Natura). Scan rate 0.1 V·s^−1^.

In the case of CV, the measurements were recorded in the potential field of −0.4 V and +0.7 V. The kinetics of the redox processes were achieved through registering the CVs with scan rates of 0.1 V·s^−1^, using 3 different quantities from each product: 0.0150 g, 0.0350 g, and 0.0550 g (Figure 8).

In order to avoid the interference of other compounds in food supplements, the current corresponding to specific detection potential for chlorogenic acid, i.e., 0.189 V, was taken into account (Table 3). To calculate the values reported in Table 7, the dilutions and the amount of food supplement used in the analysis were also taken into account.

The occurrence of an anodic peak and of a cathodic peak corresponding to the presence of CGA in real samples may be noticed in all three CVs. The responses obtained by GPH-GNP-SPE indicate that the modified sensor had good selectivity and sensitivity, appropriate for detecting CGA in nutraceutical products.

To validate the voltammetric method, FTIR spectrometric analyses were carried out for the same samples. The samples were prepared through homogenization with potassium bromide, without other stages of pre-treatment being necessary. All the experiments were carried out thrice. The wave number corresponding to the C=O group was 1680 cm^−1^ ([52] p. 1,794,427), the respective band being identified in all the samples analyzed. Consequently, absorbance for the standard sample and for the samples from food supplements was measured at this wave number. Based on the results obtained, the CGA concentrations in nutraceutical products were calculated, as shown in Table 7.

**Figure 9:**
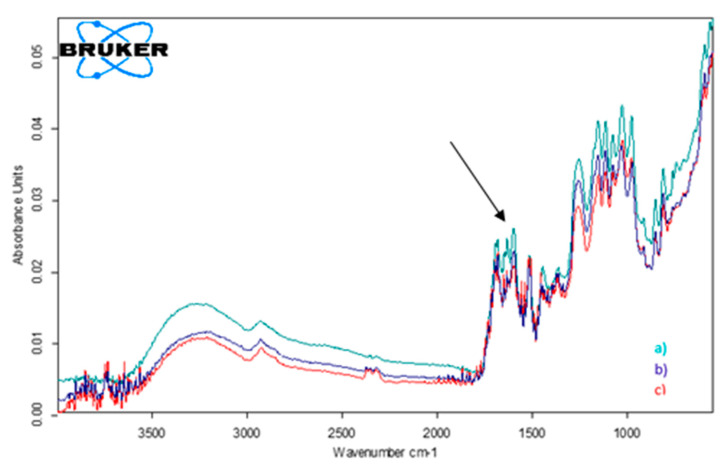
FTIR normalized spectra of (**a**) Green Coffee Extract (Rotta Natura); (**b**) Green Coffee (Pro Natura); (**c**) Green Coffee Fit (Pro Natura).

Figure 9 illustrates the FTIR spectra of the samples from the three products studied—namely, Green Coffee Extract (Rotta Natura), Green Coffee (Pro Natura), and Green Coffee Fit (Pro Natura).

### 2.6. Stability, Repeatability and Interference Studies

The stability for GPH-GNP-SPE was evaluated by carrying out 50 measurements using a 10^−3^ M CGA solution and cyclic voltammetry. The results obtained highlighted very small differences between the anodic currents, thus confirming the stability of the sensor used in the electroanalysis.

The tests for the repeatability study were carried out in a 50 µM CGA–10^−1^ M PBS solution. The value of the relative deviation standard (RDS) for the anodic peak identified for the seven consecutive determinations in the same solution did not surpass the value of 2.5%.

In optimum experimental conditions, the effect of various possible interferents under the form of organic compounds such as L-ascorbic acid, ferulic acid, and vanillic acid on CGA quantification was evaluated. The method used was cyclic voltammetry. The tolerance limit was defined as the maximum concentration of the interference compounds, which prompted a relative error of ±5% in the CGA quantitative determination. The results obtained indicate that the peaks due to the CGA presence did not change significantly on adding interferents. This is shown in Table 8.

The tolerance limit was calculated, with the following results: 5 × 10^−5^ M for the L-ascorbic acid, and 2 × 10^−5^ M for the ferulic and vanillic acids.

It may be concluded that the GPH-GNP-SPE sensor has an adequate selectivity for detecting CGA in multicomponent solutions.

## 3. Materials and Methods

### 3.1. Chemical Substances and Solutions

The reagents employed were achieved from Sigma-Aldrich (St. Louis, MO, USA), being used without any supplementary purification: potassium chloride, potassium ferrocyanide, sodium diphosphate, and phosphoric acid. During the preliminary experiments, the following potassium ferrocyanide solutions were used: 10^−3^ M K_4_[Fe(CN)_6_] and 10^−1^ M phosphate buffer solution.

To prepare the 10^−1^ M phosphate buffer solution (PBS) with pH = 7, disodium phosphate and phosphoric acid dissolved in ultrapure water obtained from the Milli-Q system (Millipore, Bedford, MA, SUA) was used. To verify the pH of the solution, a pH-meter was used (WTW Instruments, Weilheim, Germany).

The CGA (analytical purity) used in the electroanalytical studies was purchased from Sigma-Aldrich. To obtain the stock solution of 10^−3^ M CGA, the adequate quantities of CGA were dissolved in PBS solution (pH = 7).

The phenolic compounds used in the interference studies—namely, ferulic acid and vanillic acid—were purchased from Sigma-Aldrich, and L-ascorbic acid was purchased from Riedel-de-Haen (Seelze, Germany).

### 3.2. Electrochemical Cell

All electrochemical experiments were carried out using an electrochemical cell with a capacity of 50 mL (Princeton Applied Research, Oak Ridge, TN, USA), containing a system with three electrodes: the reference electrode—Ag/AgCl, a platinum wire as a counter electrode, and the working electrode. The working electrodes were carbon screen-printed electrodes (C-SPEs) (Dropsense, Llanera, Austurias, Spain) modified either with graphene or with graphene and gold nanoparticles. All measurements were carried out at room temperature.

### 3.3. Equipment

To characterize and optimize the signals of the electrodes and, at the same time, to analyze nutraceutical products, an EG&G potentiostat/galvanostat (Princeton Applied Research, Oak Ridge, TN, USA) controlled by EC-Lab Express application software that operated in Windows was used. In order to analyze and interpret the results obtained, the following programs were also used: Origin and Microsoft Excel.

To dissolve the compounds and homogenize the solutions, an Elmasonic S10H ultrasound bath (Elma Schmidbauer GmbH, Singen, Germany) was used. Weighing the substances was done at an AS 60/220.R2 analytical scale (SC Partner Corporation SRL, Bucharest, Romania).

Cyclic voltammetry was the detection technique used, and the optimal potential range was optimized for the analyzed solutions. The scan rate was 0.1 V·s^−1^, except for the studies on the influence of scan rate on the sensor response.

In order to obtain the FTIR spectra, we used the Bruker ALPHA FT-IR spectrometer (BrukerOptik GmbH, Ettlingen, Germany), connected to the OPUS software (BrukerOptik GmbH, Ettlingen, Germany), in the range of 4000–500 cm^– 1^ (32 scans, 4 cm^−1^ resolution) in the attenuated total reflectance mode (ATR). The ATR ZnSe crystal was wiped clean with ultrapure water and isopropanol between measurements. The background was the spectrum obtained in the air (ATR ZnSe crystal).

### 3.4. Real Sample Analysis

Three products were analyzed: Green Coffee Extract (Rotta Natura), Green Coffee (Pro Natura), and Green Coffee Fit (Pro Natura).

Green Coffee Extract (Rotta Natura) contains extracts of green coffee (Coffea arabica), which ensures the intensification of the metabolism. Thus, the product is indicated for reducing corporal mass, reducing cellulite, and supporting people suffering from diabetes.

Green Coffee Product (Pro Natura) contains extracts from green coffee beans. The content of this product has antioxidation properties, facilitating the neutralization of free radicals from the body and contributing to a normal energetic metabolism.

The ingredients of the Green Coffee Fit product (Pro Natura) are extract of green coffee beans with 50% chlorogenic acids, gelatin (capsule), green tea leave powder, hydroalcoholic extract 1:1 of Guarana beans based on 15% maltodextrin and 5% caramel pigment, and fruit powder from bitter orange. Due to this composition, the product is a powerful antioxidant, which may help neutralize free radicals from the body and help in fighting against physical and mental stress. At the same time, it contributes to accelerating the metabolism, facilitating the burning of subcutaneous body fat deposits.

To carry out electrochemical studies, the content of a capsule from each product was weighed and dissolved in 50 mL of PBS solution (pH = 7). The ultrasound bath was used to homogenize the content, and the insoluble fraction was separated by means of filtration.

## 4. Conclusions

Developing reliable, rapid, sensitive, and simple methods for determining and quantifying CGA is of great interest. Consequently, the present paper describes the use of various voltammetric sensors based on simple screen-printed carbon electrodes, modified with graphene on the one hand and with graphene and gold nanoparticles on the other, for the analysis and determination of CGA. Using cyclic voltammetry as method detection, very good results were obtained, with applicability in laboratory practice.

Of the three electrodes, GPH-GNP-SPE proved to have a very good sensitivity and a very low limit of detection for CGA. The analytical method may be successfully applied in determining CGA from real samples.

The results obtained with GPH-GNP-SPE are very close to those obtained through standard methods of analysis, indicating a trust level of 99%. Techniques traditionally used for the quantification of antioxidant properties of CGA in biological samples and in natural samples have a disadvantage in that they are usually time consuming and require quite expensive equipment and pre-treatment steps prior to estimation of an analyte [53]. Compared with these methods, the one proposed by us has a series of advantages, including reduced time in sample analysis, very good accuracy, small quantity of samples necessary, good precision, portability, and reduced costs, which make this method suitable for quality control of pharmaceuticals as well as nutraceutical products.

## Figures and Tables

**Table 1:** The main features of electrodes obtained by cyclic voltammetry.

Sensor	Ipa ^1^ (µA)	Ipc ^2^ (µA)	Ipc/Ipa	Epa ^3^ (V)	Epc ^4^ (V)	E_1/2_ ^5^ (V)	ΔE ^6^ (V)
C-SPE	23.88	–26.04	1.09	0.447	0.001	0.224	0.446
GPH-SPE	36.59	–40.51	1.10	0.286	0.159	0.222	0.127
GPH-GNP-SPE	81.42	–87.27	1.07	0.298	0.109	0.203	0.189

^1^ Current of the anodic peak; ^2^ current of the cathodic peak; ^3^ potential of the anodic peak; ^4^ potential of the cathodic peak; ^5^ half-wave potential; ^6^ ΔE = Epa − Epc.

**Table 2:** Ipa vs. v^1/2^ linear regression equations, determination coefficients, active surface areas, geometrical areas, and the roughness factors of all the SPEs studied.

Electrode	I_pa_ vs. v^1/2^	R^2^	Active Aria (cm^2^)	Geometrical Area (cm^2^)	Roughness Factor
C-SPE	I_pa_ (A) = 6.95 × 10^−5^ v^1/2^ (V·s^−1^)^1/2^ + 2.00 × 10^−6^	0.9999	0.0973	0.1256	0.7746
GPH-SPE	I_pa_ (A)= 2.24 × 10^−4^ v^1/2^ (V·s^−1^)^1/2^ – 4.27 × 10^−5^	0.9935	0.3145		2.4976
GPH-GNP-SPE	I_pa_ (A) = 2.64 × 10^−4^ v^1/2^ (V·s^−1^)^1/2^ – 3.31 × 10^−6^	0.9996	0.3699		2.9450

**Table 3:** The values of the parameters obtained from the CVs of all the electrodes immersed in 10^−3^ M CGA solution (the electrolyte support was 10^−1^ M PBS of pH = 7).

Electrode	Epa (V)	Ipa (µA)	Epc (V)	Ipc (µA)	Ipc/Ipa	ΔE (V)
C-SPE	0.279	21.08	0.042	–13.17	0.62	0.237
GPH-SPE	0.197	53.50	0.114	–36.69	0.68	0.083
GPH-GNP-SPE	0.189	43.23	0.113	–28.01	0.65	0.076

**Table 4:** The linear fitting equations (Ipa vs. v^1/2^), R^2^, and D of CGA.

Electrode	I_pa_ vs. v^1/2^	R^2^	D (cm^2^·s^−1^)
C-SPE	I_pa_ (A) = 4.9004 × 10^−5^ v^1/2^ (V·s^−1^)^1/2^ + 4.2335 × 10^−6^	0.9894	7.08 × 10^−9^
GPH-SPE	I_pa_ (A) = 3.0071 × 10^−4^ v^1/2^ (V·s^−1^)^1/2^ − 4.8041 × 10^−5^	0.9916	3.15 × 10^−8^
GPH-GNP-SPE	I_pa_ (A) = 2.5959 × 10^−4^ v^1/2^ (V·s^−1^)^1/2^ − 4.5262 × 10^−5^	0.9961	3.17 × 10^−8^

**Table 5:** LOD and LOQ values for CGA detection for each of the three sensors.

Sensor	LOD (M)	LOQ (M)
C-SPE	6.50 × 10^−7^	2.16 × 10^−6^
GPH-SPE	0.73 × 10^−7^	2.45 × 10^−6^
GPH-GNP-SPE	0.62 × 10^−7^	1.94 × 10^−7^

**Table 6:** Sensitive materials, detection techniques, and detection limits (LODs) of the main voltammetric sensors used for the detection of CGA.

Sensitive Material	Detection Technique	LOD (µmol·L^−1^)	Reference
GO/SPCEs ^1^	UV-Vis spectroscopy	0.16	[46]
GR-MWNTs/GCE ^2^	Capillary electrophoresis with chemiluminescence	0.5	[47]
MWCNTs/SPE ^3^	DPV	0.12	[48]
MIS/Au electrode ^4^	DPV	0.15	[22]
PASA/GCE ^5^	CV	0.04	[44]
Ir-BMI.PF_6_^6^	SWV	0.09	[18]
IL/DMC/PE ^7^	SWV	0.01	[49]

DPV, differential pulse voltammetry; SWV, square-wave voltammetry; CV, cyclic voltammetry; ^1^ graphene oxide modified electrochemically pre-anodized screen-printed carbon electrodes; ^2^ graphene dispersed multi-walled carbon nanotubes composite modified glassy carbon electrode; ^3^ multi-walled carbon nanotubes modified screen-printed electrode; ^4^ molecularly imprinted siloxane (MIS) film, prepared by sol–gel process, onto Au bare electrode; ^5^ poly (aminosulfonic acid) modified glassy carbon electrode; ^6^ 1-n-butyl-3-methylimidazolium hexafluorophosphate containing dispersed iridium nanoparticles; ^7^ highly defective mesoporous carbon—ionic liquid paste electrode.

**Table 7:** CGA content determined in three food supplements by using voltammetric and spectrophotometric method.

Food Supplement	Voltammetric Method c% CGA	FTIR Method c% CGA
Green Coffee Extract (Rotta Natura)	4.43 ± 0.11	4.83 ± 0.12
Green Coffee (Pro Natura)	4.38 ± 0.11	4.62 ± 0.12
Green Coffee Fit (Pro Natura)	5.86 ± 0.12	6.43 ± 0.11

**Table 8:** The interference of some chemically related species on the quantitative determination of CGA.

Interfering Compound	Concentration of the Interfering Chemically Related Species (M)	Recovery %	RSD %
L-ascorbic acid	10^−5^ M	102.09	1.46
Ferulic acid	10^−5^ M	104.19	2.90
Vanillic acid	10^−5^ M	103.80	2.64

## Data Availability

The authors confirm that the data supporting the findings of this study are available within the article.

## References

[1] Oliver P., Villem A. (2017). Phenolic Compounds: Structure, Uses and Health Benefits.

[2] Teixeira J., Gaspar A., Garrido E.M., Garrido J., Borges F. (2013). Hydroxycinnamic Acid Antioxidants: An Electrochemical Overview. BioMed Res. Int..

[3] Nguyen T., Sherratt P.J., Pickett C.B. (2003). Regulatory mechanisms controlling gene expression mediated by the antioxidant response element. Annu. Rev. Pharmacol. Toxicol..

[4] Crupi P., Bleve G., Tufariello M., Corbo F., Clodoveo M.L., Tarricone L. (2018). Comprehensive identification and quantification of chlorogenic acids in sweet cherry by tandem mass spectrometry techniques. J. Food Compos. Anal..

[5] Zhang B., Yang R., Zhao Y., Liu C.-Z. (2008). Separation of chlorogenic acid from honeysuckle crude extracts by macroporous resins. J. Chromatogr. B Analyt. Technol. Biomed. Life Sci..

[6] Dai C.-Y., Gao X.-Y., Tang B., Fu Y., Liu H.-A. (2010). Determination of the contents of chlorogenic acid and phillyrin of Shuanghuanglian oral fluid using NIRS. Guang Pu Xue Yu Guang Pu Fen Xi Guang Pu.

[7] Upadhyay R., Rao L.J.M. (2013). An Outlook on Chlorogenic Acids—Occurrence, Chemistry, Technology, and Biological Activities. Crit. Rev. Food Sci. Nutr..

[8] Tan L., Xin S., Guangxin Y. Rapid and simultaneous determination of rutin, chlorogenic acid and quercetin in mulberry folium leaf by capillary electrophoresis. Proceedings of the 2011 International Conference on Human Health and Biomedical Engineering.

[9] Li Z., Huang D., Tang Z., Deng C., Zhang X. (2010). Fast determination of chlorogenic acid in tobacco residues using microwave-assisted extraction and capillary zone electrophoresis technique. Talanta.

[10] Hu F., Deng C., Liu Y., Zhang X. (2009). Quantitative determination of chlorogenic acid in Honeysuckle using microwave-assisted extraction followed by nano-LC-ESI mass spectrometry. Talanta.

[11] Wang X., Wang J., Yang N. (2007). Chemiluminescent determination of chlorogenic acid in fruits. Food Chem..

[12] Es’haghi Z., Golsefidi M.A., Saify A., Tanha A.A., Rezaeifar Z., Alian-Nezhadi Z. (2010). Carbon nanotube reinforced hollow fiber solid/liquid phase microextraction: A novel extraction technique for the measurement of caffeic acid in Echinacea purpurea herbal extracts combined with high-performance liquid chromatography. J. Chromatogr. A.

[13] Apetrei I., Apetrei C. (2019). Development of a Novel Biosensor Based on Tyrosinase/Platinum Nanoparticles/Chitosan/Graphene Nanostructured Layer with Applicability in Bioanalysis. Materials.

[14] Barroso M.F., de-los-Santos-Álvarez N., Delerue-Matos C., Oliveira M.B.P.P. (2011). Towards a reliable technology for antioxidant capacity and oxidative damage evaluation: Electrochemical (bio)sensors. Biosens. Bioelectron..

[15] Bard A.J., Faulkner L.R. (2001). Electrochemical Methods: Fundamentals and Applications.

[16] Yola M.L., Atar N., Üstündağ Z., Solak A.O. (2013). A novel voltammetric sensor based on p-aminothiophenol functionalized graphene oxide/gold nanoparticles for determining quercetin in the presence of ascorbic acid. J. Electroanal. Chem..

[17] Delgado A.M., Issaoui M., Chammem N. (2019). Analysis of Main and Healthy Phenolic Compounds in Foods. J. AOAC Int..

[18] Fernandes S.C., Moccelini S.K., Scheeren C.W., Migowski P., Dupont J., Heller M., Micke G.A., Vieira I.C. (2009). Biosensor for chlorogenic acid based on an ionic liquid containing iridium nanoparticles and polyphenol oxidase. Talanta.

[19] Mello L.D., Sotomayor M.D.P.T., Kubota L.T. (2003). HRP-based amperometric biosensor for the polyphenols determination in vegetables extract. Sens. Actuators B Chem..

[20] Moccelini S.K., Spinelli A., Vieira I.C. (2008). Biosensors based on bean sprout homogenate immobilized in chitosan microspheres and silica for determination of chlorogenic acid. Enzyme Microb. Technol..

[21] De Carvalho M.L., Santhiago M., Peralta R.A., Neves A., Micke G.A., Vieira I.C. (2008). Determination of chlorogenic acid in coffee using a biomimetic sensor based on a new tetranuclear copper(II) complex. Talanta.

[22] Santos W.d.R., Santhiago M., Yoshida I.V.P., Kubota L.T. (2011). Novel electrochemical sensor for the selective recognition of chlorogenic acid. Anal. Chim. Acta.

[23] Rawson F.J., Jackson S.K., Hart J.P. (2010). Voltammetric Behavior of DNA and Its Derivatives Using Screen Printed Carbon Electrodes and Its Possible Application in Genotoxicity Screening. Anal. Lett..

[24] Honeychurch K.C., Hart J.P., Cowell D.C., Arrigan D.W.M. (2001). Voltammetric studies of lead at calixarene modified screen-printed carbon electrodes and its trace determination in water by stripping voltammetry. Sens. Actuators B Chem...

[25] Tan F., Metters J.P., Banks C.E. (2013). Electroanalytical applications of screen printed microelectrode arrays. Sens. Actuators B Chem..

[26] Mincu N.-B., Lazar V., Stan D., Mihailescu C.M., Iosub R., Mateescu A.L. (2020). Screen-Printed Electrodes (SPE) for In Vitro Diagnostic Purpose. Diagnostics.

[27] Kerman K., Saito M., Tamiya E., Yamamura S., Takamura Y. (2008). Nanomaterial-based electrochemical biosensors for medical applications. TrAC Trends Anal. Chem..

[28] Martínez-Periñán E., Gutiérrez-Sánchez C., García-Mendiola T., Lorenzo E. (2020). Electrochemiluminescence Biosensors Using Screen-Printed Electrodes. Biosensors.

[29] Zhang W., Wang R., Luo F., Wang P., Lin Z. (2020). Miniaturized electrochemical sensors and their point-of-care applications. Chin. Chem. Lett..

[30] Wang J., Pedrero M., Pamidi P.V.A., Cai X. (1995). Metal-dispersed screen-printed carbon electrodes. Electroanalysis.

[31] Apetrei I.M., Apetrei C. (2018). A modified nanostructured graphene-gold nanoparticle carbon screen-printed electrode for the sensitive voltammetric detection of rutin. Measurement.

[32] Torres-Rivero K., Florido A., Bastos-Arrieta J. (2021). Recent Trends in the Improvement of the Electrochemical Response of Screen-Printed Electrodes by Their Modification with Shaped Metal Nanoparticles. Sensors.

[33] Zhang W., Zhu S., Luque R., Han S., Hu L., Xu G. (2016). Recent development of carbon electrode materials and their bioanalytical and environmental applications. Chem. Soc. Rev..

[34] Ambaye A.D., Kefeni K.K., Mishra S.B., Nxumalo E.N., Ntsendwana B. (2021). Recent developments in nanotechnology-based printing electrode systems for electrochemical sensors. Talanta.

[35] Hamblin M.R., Avci P. (2015). Applications of Nanoscience in Photomedicine.

[36] Pingarrón J.M., Yáñez-Sedeño P., González-Cortés A. (2008). Gold nanoparticle-based electrochemical biosensors. Electrochim. Acta.

[37] Chauhan P., Annu, Raja A.N., Jain R. (2020). Nanogold modified glassy carbon sensor for the quantification of phytoestrogenchlorogenic acid. Surf. Interfaces.

[38] Forzato C., Vida V., Berti F. (2020). Biosensors and Sensing Systems for Rapid Analysis of Phenolic Compounds from Plants: A Comprehensive Review. Biosensors.

[39] Kaya S.I., Karabulut T.C., Kurbanoglu S., Ozkan S.A. (2020). Chemically Modified Electrodes in Electrochemical Drug Analysis. Curr. Pharm. Anal..

[40] Namazian M., Zare H.R. (2005). Electrochemistry of chlorogenic acid: Experimental and theoretical studies. Electrochim. Acta.

[41] Zare H.R., Nasirizadeh N., Ajamain H., Sahragard A. (2011). Preparation, electrochemical behavior and electrocatalytic activity of chlorogenic acid multi-wall carbon nanotubes as a hydroxylamine sensor. Mater. Sci. Eng. C.

[42] Bounegru A.V., Apetrei C. (2020). Carbonaceous Nanomaterials Employed in the Development of Electrochemical Sensors Based on Screen-Printing Technique—A Review. Catalysts.

[43] Apetrei I.M., Apetrei C. (2016). Voltammetric determination of melatonin using a graphene-based sensor in pharmaceutical products. Int. J. Nanomed..

[44] Chao M., Ma X. (2014). Voltammetric determination of chlorogenic acid in pharmaceutical products using poly(aminosulfonic acid) modified glassy carbon electrode. J. Food Drug Anal..

[45] Armbruster D.A., Pry T. (2008). Limit of blank, limit of detection and limit of quantitation. Clin. Biochem. Rev..

[46] Zhang L., Li Y., Zhang L., Li D., Karpuzov D., Long Y. (2011). Electrocatalytic Oxidation of NADH on Graphene Oxide and Reduced Graphene Oxide Modified Screen-Printed Electrode. Int. J. Electrochem. Sci..

[47] Ye L., Xiang M., Zhang Y., Luo L., Gao Y., Yu J., Cha J. (2013). A Novel Electrochemical Method for Sensitive Detection of Anticancer Drug Picoplatin with Graphene Multi-walled Carbon Nanotubes Modified Glassy Carbon Electrode. Int. J. Electrochem. Sci..

[48] Ma X., Yang H., Xiong H., Li X., Gao J., Gao Y. (2016). Electrochemical Behavior and Determination of Chlorogenic Acid Based on Multi-Walled Carbon Nanotubes Modified Screen-Printed Electrode. Sensors.

[49] Mohammadi N., Najafi M., Adeh N.B. (2017). Highly defective mesoporous carbon—Ionic liquid paste electrode as sensitive voltammetric sensor for determination of chlorogenic acid in herbal extracts. Sens. Actuators B Chem..

[50] Albu C., Eremia S.A.V., Veca M.L., Avram A., Popa R.C., Pachiu C., Romanitan C., Kusko M., Gavrila R., Radoi A. (2019). Nano-crystalline graphite film on SiO_2_: Electrochemistry and electro-analytical application. Electrochim. Acta.

[51] Council of Europe, European Pharmacopoeia Commission, European Directorate for the Quality of Medicines & Healthcare (2010). European Pharmacopoeia.

[52] Chlorogenic Acid—Compound Summary. https://pubchem.ncbi.nlm.nih.gov/compound/1794427.

[53] Singh K., Jadon N., Jain R. (2018). Synergistic effect of 1-butyl-2,3-dimethylimidazolium bis (trifluoromethanesulfonyl) imide and titanium oxide on the redox behaviour of flunarizine in solubilized media. Colloids Surf. B Biointerfaces.

